# The role of implementation organizations in scaling evidence-based psychosocial interventions

**DOI:** 10.1186/s13012-023-01280-5

**Published:** 2023-06-22

**Authors:** Margaret E. Crane, Philip C. Kendall, Bruce F. Chorpita, Matthew R. Sanders, Allen R. Miller, Carolyn Webster-Stratton, Jenna McWilliam, Judith S. Beck, Ceth Ashen, Dennis D. Embry, John A. Pickering, Eric L. Daleiden

**Affiliations:** 1 Department of Psychology, Temple University, Weiss Hall, 1701 N 13th St, Philadelphia, PA 19122 USA; 2 Department of Psychiatry, New York Presbyterian-Weill Cornell Medicine, 425 E 61st St, New York, NY 10065 USA; 3 Department of Psychology, University of California Los Angeles, 502 Portola Plaza, Los Angeles, CA 90095 USA; 4 PracticeWise, LLC, 410 Coach Rd, Satellite Beach, FL 32937 USA; 5 Parenting and Family Support Centre, School of Psychology, The University of Queensland, St Lucia, QLD 4067 Australia; 6 Beck Institute for Cognitive Behavior Therapy, 1 Belmont Ave #700, Bala Cynwyd, PA 19004 USA; 7 Incredible Years Inc, 1411 8th Avenue, West Seattle, WA 98119 USA; 8 Triple P International, 11 Market St N, Indooroopilly, QLD 4068 Australia; 9 C. Ashen Consulting, 222 North Canon Dr. Ste 205, Beverly Hills, CA 90210 USA; 10 PAXIS Institute, PO Box 31205, Tucson, AZ 85751 USA; 11 Evidn, 315 Brunswick St, Fortitude Valley, QLD 4006 Australia

**Keywords:** Commercialization, Industry, Purveyor, Intermediary, Dissemination, Implementation, Evidence-based intervention, Stages of Small Business Growth

## Abstract

**Background:**

To bring evidence-based interventions (EBIs) to individuals with behavioral health needs, psychosocial interventions must be delivered at scale. Despite an increasing effort to implement effective treatments in communities, most individuals with mental health and behavioral problems do not receive EBIs. We posit that organizations that commercialize EBIs play an important role in disseminating EBIs, particularly in the USA. The behavioral health and implementation industry is growing, bringing the implementation field to an important inflection point: how to scale interventions to improve access while maintaining EBI effectiveness and minimizing inequities in access to psychosocial intervention.

**Main body:**

We offer a first-hand examination of five illustrative organizations specializing in EBI implementation: Beck Institute for Cognitive Behavioral Therapy; Incredible Years, Inc.; the PAXIS Institute; PracticeWise, LLC; and Triple P International. We use the Five Stages of Small Business Growth framework to organize themes. We discuss practical structures (e.g., corporate structures, intellectual property agreements, and business models) and considerations that arise when trying to scale EBIs including balancing fidelity and reach of the intervention. Business models consider who will pay for EBI implementation and allow organizations to scale EBIs.

**Conclusion:**

We propose research questions to guide scaling: understanding the level of fidelity needed to maintain efficacy, optimizing training outcomes, and researching business models to enable organizations to scale EBIs.

Contributions to the literature

- This article provides first-hand reflections on practical and ethical issues that come with commercializing the dissemination and implementation of psychosocial interventions.
- This article uses the Five Stages of Small Business Growth framework to discuss implementation-related considerations throughout the growth of implementation organizations.
- This article discusses how to balance cost to payors and consumers with the need for a financially sustainable business.
- This article reviews the potential trade-offs between fidelity and reach of evidence-based interventions.
- This article considers strategies to manage conflict of interest when conducting research with commercial partners.

## Background

An evidence-based intervention (EBI) must be implemented at scale to make a meaningful public health impact [1]. Fewer than 50% of adults and 20% of youth with mental health disorders receive any treatment [2, 3], and far fewer receive EBIs [4]. Three pillars support EBI impact: (1) research evidence supporting effectiveness, (2) demand for the intervention, and (3) capacity to implement the intervention at scale [5]. Research has yielded an array of EBIs [6], the field has partially succeeded at increasing demand for services [7], and yet insufficient capacity exists for implementing EBIs in the community.

Behavioral health and implementation are growth industries [7]. Corporations and entrepreneurs are entering the behavioral health industry, especially in the USA [8]. Behavioral health companies received over US $4.82 billion in private capital in 2021, representing a fourfold increase in private funding since 2019 [9]. Mental health teletherapy (e.g., Talkspace, BetterHelp, Lyra) and smartphone apps (e.g., Headspace and Calm) are increasing in popularity [10, 11]. As of 2021, seven mental health companies are now considered “unicorns” (companies valued at more than US $1 billion) [7], demonstrating potential to scale behavioral health practices. To achieve high-quality services for all, EBI implementation must keep pace with the scale of service growth. The implementation field is at an important juncture, prompting this discussion on scaling while promoting equitable access to EBIs.

### Dissemination and implementation organizations

Organizations that specialize in EBI dissemination and implementation (D&I) include the following: intermediary and purveyor organizations (IPOs) [12], Centers for Excellence [13], and community-academic partnerships [14]. IPOs, referred to as “bridging factors” in implementation efforts, bridge the gaps between societal contexts, organizations, and EBIs [15, 16]. There is considerable overlap in IPO activities [12]; both organizations implement EBIs in the community [17]. Purveyor organizations work to effectively implement specific program(s) [18], while intermediary organizations implement many EBIs by building capacity within an agency or system, such as through workforce development and standard setting [12]. Intermediary organizations often operate locally, while purveyor organizations operate nationally/internationally. IPOs work together or separately, as well as with local providers, to implement EBIs in a local context. The logic models of IPOs posit that through the delivery of training programs and ongoing implementation supports, providers gain knowledge in delivering a program, providers deliver the program (ideally with fidelity), and client outcomes improve [19, 20]. This focus differentiates IPOs from companies that employ or contract providers to provide services (e.g., Equip, Lyra).

IPOs have grown in number and revenue in the USA [12, 19]. More researchers and practitioners are starting or joining organizations with a mission to increase access to EBIs (e.g., Single-Session Support Solutions; Be Braver; Lyssn; the Center for Implementation; Equip). A 2019 study estimated the annual revenue across 11 nonprofit IPOs at almost US $2 billion, suggesting that IPOs play a significant role in the implementation industry [19]. The implementation industry is in a period of both proliferation (i.e., new companies being created) and consolidation. Companies, such as Empower Community Care, have begun acquiring other purveyor organizations (e.g., Multisystemic Therapy Services, Incredible Years, Inc., Functional Family Therapy), suggesting that the implementation industry is entering a phase of consolidation of purveyor organizations. Understanding the strategies of successful IPOs can guide more efficient design of future implementation organizations.

A few studies have described the implementation strategies and frameworks IPOs use to implement EBIs [12, 19–22]. IPO primary functions are staff training and consultation support, quality assurance (i.e., fidelity monitoring), resource mobilization, facilitating connections among partners, providing implementation support, policy and systems development, advocacy and visibility, and best practice model development [12, 21–23]. McWilliam et al. [20] described Triple P International’s five implementation phases as engagement, commitment and contracting, implementation planning, training and accreditation, and implementation and maintenance. Proctor et al. found that IPOs use an average of 32 implementation strategies [19]. Education, planning, and quality improvement were most frequently endorsed, while financial, restructuring, and quality management were less frequently endorsed [19]. Finally, Franks and Bory [17] discussed factors that contribute to the success of developing an intermediary organization, including a favorable political climate, access to technical assistance, and stable funding (often government).

To advance analysis beyond identification of strategies, the present paper discusses the opportunities and threats that affect IPOs’ decision-making as they grow. We organize themes using the Five Stages of Small Business Growth framework [24]; stages include existence, survival, success, take-off, and maturity. Through each of these stages, businesses use various strategies to generate profit both to stabilize and grow their business, and to achieve their mission. This paper discusses practical decisions implementation organizations make while aiming to increase access to EBIs.

## Method

To elucidate practical issues that arise as IPO’s scale, we provide first-hand reflections of five implementation organizations (EBI that the company scales is in parenthesis): (1) Beck Institute (Beck model of cognitive behavioral therapy); (2) Incredible Years, Inc. (Incredible Years), purchased by Empower Community Care in 2022; (3) PAXIS Institute (PAX Good Behavior Game); (4) PracticeWise, LLC (Modular Approach to Treatment of Children with Anxiety, Depression, or Conduct Problems [MATCH], Managing and Adapting Practice [MAP], PracticeWise Evidence-Based Services, Core Elements of Family Therapy); and (5) Triple P International (Triple P-Positive Parenting Program). The five organizations were selected due to their wide reach: their operating budgets are in the top quartile of IPOs [12], and the number of clinicians trained is in the top 10% of IPOs [19]. Table 1 provides an overview of these organizations; Table 2 summarizes their D&I activities. These organizations can be considered an IPO.^1^ The organizations primarily disseminate EBIs for youth mental health; the Beck Institute also disseminates EBIs for adults. Four out of five organizations are based in the USA, and one is based in Australia. All five organizations operate internationally and have trained providers in over 120 countries. The considerations discussed in this paper may be most relevant for countries in which businesses – rather than universities, governments, or nongovernmental organizations – conduct the majority EBI trainings.

**Table 1:** Overview of organizations reviewed as of 2022

Topic	Beck Institute	Incredible Years, Inc	PAXIS Institute	PracticeWise	Triple P International
**Organization background**					
Website	beckinstitute.org	incredibleyears.com	paxis.org	practicewise.com	triplep.net
Programs/services offered	Training and dissemination of Beck model of CBT, online CBT resources, provide CBT	Training and dissemination of Incredible Years programs	Training and dissemination of PAX Good Behavior Game	Information services; resource subscriptions (e.g., MAP, PWEBS, MATCH, CEFT), consultation, credentialing	Implementation support, program resources, and training for the Triple P-Positive Parenting Programs
Number of paid staff members	65	4	70	49	120
Estimated number of providers trained	40,000	72,500	60,000	39,500 registered; 7100 credentialed	100,000
Number of trainers	54	145 (10 trainers, 59 mentors who can deliver workshops, and 167 peer coaches)	45	550 (32 training professionals; 317 agency supervisors; 205 instructors)	98 trainers
US states and countries with trained providers	50 US states; 130 countries	50 US states; 33 countries	38 US states, 6 countries	Registered: 50 US states, 130 countries; credentialed: 28 states; 7 countries	38 US states; 72 countries
**Corporate structures**					
Profit structure	501(c)3	For profit	For profit	For profit	For profit and Certified B Corp®
Board of directors/advisory board	Yes/yes	No/no	No/no	Yes/yes	No/no
Number of program developers	2	1	11	30	9
Program developer(s) role in organization	Various: founder and president emeritus; president	Founder/president	Founder/president/CEO	Various: unaffiliated, consultant, employee, board member, founder	Various: consultants, trainers
Program developer university affiliation	Professor at University of Pennsylvania	Past: graduate student, professor; present: professor emeritus	Co-investigator at Johns Hopkins	Various: professor, clinical affiliate, none	Professor at The University of Queensland
Student opportunities	Yes	Yes	Yes	Yes	Yes
COI protocol	External experts to design RCTs, data management, data analysis; COI statements on publications	External data manager; financial disclosures yearly to University of Washington; COI statements on publications	Independent scientists with explicit agreements to publish regardless of outcomes	Minimize or eliminate role in data collection and analysis; COI statements on publications	External data management and data analysis; standard COI statement on publications
**Intellectual property**					
Ownership of intellectual property rights	Beck Institute for Cognitive Behavior Therapy	Carolyn Webster-Stratton	Dennis Embry	Various from proprietary to diverse licensing arrangements that differ by products and services	The University of Queensland
**Matters of pricing**					
Main revenue	Trainings	Trainings and product sales	Training, materials and other supports	Information services, training, and consultation	Training and sale of program resources
Main revenue source	Not-for-profit organizations	Governments contracts; health systems; universities	Schools, governments, health, and schools	Business-to-business services	Government contracts

*CBT* cognitive behavioral therapy, *COI* conflict of interest, *MATCH* modular approach to treatment of children with anxiety, depression, or conduct problems, *MAP* managing and adapting practice, *PWEBS* PracticeWise Evidence-Based Services, *CEFT* Core Elements of Family Therapy

**Table 2:** Overview of organization activities

	**Beck Institute**	**Incredible Years, Inc**	**PAXIS**	**PracticeWise**	**Triple P International**
Agency readiness measure	Interviews, focus groups, individualized assessments, and customized training plans	Launching IY programs in your organization	Depending on product/service	Varies depending on implementation model	Triple P Implementation Framework (includes facilitated organizational assessment and readiness measures)
Typical length of courses	2–3 days	3 days (online training is 15 h)	1–3 days	1–5 days	1–4 days
Consultation/supervision post-training	Yes	Yes	Yes	Yes	Yes
Provider consultation/supervision process	Recorded session review	Recorded session review	Local supervision from site-based coaches (“partners”)	Varies: performance standards, portfolio review, dashboard review	Local supervision; additional supervision provided as needed
Consultation required for accreditation	Minimum of 10, 1-h calls over 6 months (1 per client)	Amount varies and continues until video of session passed and protocols approved	No	Amount varies by credential: range from 6 to 25 h over 6–12 months	No
Provider accreditation requirement	No	Yes	No	No	Yes
Requirement for provider accreditation	Training attendance; supervision sessions with review of recorded sessions; scored by the cognitive therapy rating scale	Training attendance; lead minimum of two groups: client evaluations and self- and peer-rated fidelity checklists; video review	Training attendance and performance for some functions	Varies by credential; performance standards; portfolio evaluation; case material review	Training attendance; satisfactory demonstration of key competencies; completion of a quiz
Train-the-trainer model	Yes	Yes	No	Yes, multiple pathways	No
Provider conferences	Yes, but not hosted by the Beck Institute	Incredible Years mentor meeting	Summit, weekly zoom	Not routine, initiative focused	Helping Families Change Conference; regional Triple P Update conferences; master classes
Proactively contact policy makers about funding program	No	Not commonly	Yes	No	Yes
Engages with direct-to-consumer marketing	Yes (social media)	No	Yes (social media)	Yes (social media)	Yes
Systems consulting	Yes	Yes, when requested	Yes	Yes	Yes

We discuss considerations implementation organizations address across stages of business growth related to organization structures and cost of services. A survey was administered to all five organizations to elicit key themes (see Appendix). Questions inquired about organizational structures, key collaborators, balancing financial and D&I priorities, and general feedback. Co-authors from each organization submitted responses to survey items in writing. The first author grouped responses thematically and drafted the results. Based on the themes that emerged, we presented the themes according to the Five Stages of Small Business Growth framework [24]. Of note, many of the themes presented occur across each stage of growth; we discuss the theme in the phase of growth most relevant to that theme. Member checking was conducted by having all authors review the themes presented in the manuscript [25]. Based on their feedback, the first author revised the manuscript. This process continued iteratively until all authors agreed on the themes presented in this manuscript.

### Five Stages of Small Business Growth

#### Existence stage

The first stage of small business growth is existence, where a company is created to deliver a product to customers [24]. All five illustrative implementation organizations began either to create a more sustainable mechanism for disseminating their program or to enable interstate transfer of trainings and resources without bureaucratic barriers of a university setting. The activities conducted by implementation organizations are demanding and complex and typically outside university researchers’ interests or skills [26]. In most industries, there is a labor specialization between the person who invents, manufactures, distributes, markets, and provides technical assistance for a product [26]. Some argue that the lack of widespread EBI availability is due to underemphasizing dissemination and greater need for specialization [27]. The organizations reviewed reported that using professors and graduate students as trainers was insufficient to meet EBI training demands. Benefits of IPOs existing separately from universities include a lack of university bureaucracy, organizational independence, and the ability to operate more efficiently on a faster timeline and larger scale [23].

The creation of an implementation organization to disseminate EBIs is not the only model for wide-reaching D&I. EBI scale-ups can happen because of partnerships between governments, researchers, and local organizations [28]. Some behavioral health professionals are uncomfortable with commercializing psychosocial interventions [8]. Reasons against privatizing public health programs include challenges in ensuring effectiveness, equity, and trust in service quality [29]. Nonetheless, more professors may start behavioral health or implementation organizations as innovation and entrepreneurship become increasingly recognized as a metric for tenure and promotions, as more universities train staff and students to commercialize intellectual property, and as more people move from academia to industry [30, 31].

In each of the organizations reviewed, at least one of the program developers was involved in establishing the organization. Intervention developers’ roles in organizations range from having no role, to consulting roles (e.g., focusing on scientific evaluations, training, and program fidelity), and to being the organization head. Developers’ university affiliations also vary (e.g., full time, part-time, to emeritus), both between organizations and over time. When researchers work with implementation organizations, roles must be delineated. The role program developers play in organizations affects their perspective and priorities as collaborators in the organization’s implementation work.

Program developers must decide whether they want to create a new organization or to have their EBI be disseminated by an existing organization. The changing behavioral health industry led some developers to sell their organizations to larger organizations. For example, since its merger with Empower Community Care in 2022, Incredible Years operates independently; the developer is in charge of fidelity, training mentors and coaches, updating programs, and consulting on research and has reduced her time on business matters, marketing, and inventory management. The merger aims to increase the reach of program by taking advantage of economies of scale to reduce fixed costs. Although consolidation could lead to monopolization of prices, communities can benefit from having fewer IPOs to collaborate with [27].

If a founder decides to form a new organization, an early decision is whether to be a for-profit and/or a nonprofit company. The primary difference is the capital source: for-profit businesses can raise investments from private investors (with the expectation of a financial gain to the investor/s), whereas nonprofit businesses raise money from foundations, individuals, corporations, and governments (with the expectation of a social return). The Beck Institute is nonprofit, while Empower Community Care (owner of Incredible Years), the PAXIS Institute, PracticeWise, and Triple P International are for-profit. Triple P International is a Certified B Corporation®, which recognizes its commitment to conducting business in a way that creates public benefit and sustainable value, beyond seeking profit. Regardless of profit status, the mission of the five organizations is to improve the lives of people and communities using EBIs supported by an economically sustainable dissemination model. The implementation organizations reviewed can be considered social enterprises given their social mission [32].

The degree to which a board of directors and advisory boards play a role in implementation organizations’ work is an important research question. Although both boards provide strategic advice, boards of directors have fiduciary responsibilities and a greater legal status. In the USA, a board of directors is required for nonprofit corporations and is common with for-profit organizations. The Beck Institute and PracticeWise have both a board of directors and an advisory board that include members with various societal interests who advise organizations on local and global needs.

IP ownership–such as trademark (e.g., brand name, logo) and copyright (e.g., materials distributed)–needs to be established to mitigate disputes. EBIs include copyrighted materials, videos, online programs, manuals, workbooks, and handouts. IP ownership affects licensing agreement(s) required for implementation organizations. The IP owner may assign ownership or establish a licensing agreement with the organization to allow them to exercise specific rights associated with the IP (e.g., reproduce and distribute copyrighted materials). In return, the IP owner negotiates consideration for IP exchange (e.g., a sale price; royalty rate). IP owners can reserve IP rights (e.g., the right to prepare derivative works) or negotiate nonexclusive agreements (e.g., asset licensing to ongoing research). Triple P International reported that their exclusive license agreement supports quality control by having common training standards globally. Organizations may develop proprietary IP (e.g., introducing new trademarks, creating new or derivative works) to which the program developer may or may not have negotiated rights. Similarly, consideration needs to be given to whether local community organizations share copyright IP or have a royalty consideration if they are involved in cultural adaptations of programs. Copyright sharing may vary depending on the type and extent of involvement in adaptation.

Creative Commons licenses are an alternative model that provides free access to materials. From a business perspective, Creative Commons effectively trade consideration in the form of citation for limited use and reproduction of copyright. This citation may generate value (e.g., brand awareness, reputation) that may be monetized (e.g., higher salaries, consulting). However, many of the same costs are still required (e.g., creating, maintaining, updating, translating, adapting, and printing materials as well as and training and implementation support). These are “hidden” costs that rely on professionals volunteering their time or ongoing funding from universities, foundations, or grants. Open-access materials shift costs away from consumers and end users and to program creators and other funders. This shift creates a risk that programs do not evolve, or there is program drift as infrastructure is not available to support ongoing implementation, research, and development.

#### Survival stage

In the survival stage of small business growth, companies focus on the relation between revenue and expenses [24]. Revenue is necessary for a company to stay in business. Business models of implementation organizations are designed based on their goals and purpose, with considerations on who will pay for the EBI. Two models include (1) business to business (B2B)—one business selling to another (e.g., the five organizations reviewed) and (2) business to consumer (B2C)—a business selling directly to a consumer [33]. Many companies (e.g., Talkspace, BetterHelp, Headspace; Triple P International) have both B2B and B2C models. Regardless of the model, implementation organizations have a business mindset [5]. Income enables organizations to scale their business and disseminate EBIs to wider groups. The five organizations rely less on research grants and more on revenue from trainings, materials sales, and by providing consultations to agencies adopting the EBI. Potential funders include foundation grants, nonprofit organizations (e.g., schools, behavioral health organizations, hospitals), businesses (e.g., insurance companies), or the government (e.g., to train providers in a public system or schools). Some organizations, such as the Beck Institute, have an endowment. Behavioral health startups often rely on venture capital and other private funds to sustain operations until they are profitable. The five organizations described reinvest a substantial portion of profits in developing new programs, including updating and adapting materials, maintaining online programs, and creating mechanisms to increase program implementation.

Organizations use standard business practices (e.g., strategic planning, goal setting, and budgeting). From a unit economics perspective, many company costs are fixed. For example, there is a standard licensing fee, yet implementing a program in a new region often requires an initial investment to understand and adapt the program to local context. Global operations incur additional costs for legal advice, translations, transportation, and currency fluctuations. Subscription models to provide ongoing services can minimize working capital costs and increase financial stability. Multiple product types can generate resilient revenue streams to avoid unstable funding structures that can jeopardize organization sustainability and capacity to grow [23]. Diverse revenue streams can help organizations adapt to emerging research evidence. One-time costs are often easier to arrange and manage than recurring costs. It is not uncommon for organizations to receive “last minute” requests (e.g., “We have unspent funds that need to be expended by X date”). However, last minute requests are not generally a recipe for long-term success. Short-term relationships can “plant a seed,” but long-term partnerships are preferred. Many of the organizations reviewed have long-term partnerships with public mental health, education, and child welfare systems.

Should EBIs be distributed freely? A more nuanced question is as follows: who should bear EBI costs? Government initiatives or nonprofits can pay both for clinicians to be trained in EBIs (thus decreasing organizational training costs) and for clients to receive EBIs, such as in the Improving Access to Psychological Therapies initiative in the UK [34]. Absent such funding, freemium models can allow organizations to offer some services at no cost to establish a foundation for future transactions [35]. For example, PracticeWise offers some materials for free, whereas other resources require a subscription. Conversely, Triple P International post selected program materials on their provider website, which are free after providers have received training.

The five organizations reviewed are aware of the question, “How are we generating real economic value?” Asking “Is this program low cost?” is a relative question; low cost is compared to what, including doing nothing? Quantification of return on investment of training or service for the individual or organization can be challenging [36]. Training benefits can occur at the client, provider, and organizational levels [37]. For example, consider the value proposition of the PracticeWise Evidence-Based Services database. This service aids clinical decision-making by allowing providers to search a regularly updated online database with youth-specific summaries from published intervention research. By outsourcing research analysis and reporting functions to a central agency, organizations share the cost of these functions.

The implementation organizations reviewed often operate within the underfunded human and social services sectors. They are aware that cost is a barrier for providers. Due to an implementation organization’s need to be sustainable, potential consumers of services (i.e., providers, organizations) may be negatively impacted by high up-front prices driven by the cost of materials and experts to provide training and consultation. Although consultation calls increase training efficacy [38, 39], they increase the cost and provider training burden [40]. Many organizations offer flexible pricing for special needs or when services are purchased in bulk. For some programs, the contracted trainers and supervisors (i.e., not the organization) set their fee rate for training and supervision.

Cost–benefit analyses suggest that EBIs produce social benefits, decreased involvement with the criminal justice system, and increased work productivity and participation [41]. Cost-effectiveness is an important aspect of scalability [5, 42]. One study found CBT training by the Beck Institute cost US $0.18 per consumer [40]. An economic analysis of EBI cost-effectiveness for child behavioral health problems found the cost–benefit ratio per participant served was positive for Incredible Years (US $5.65), MATCH (US $7.64), the Triple P system (US $9.71), and the Good Behavior Game (US $62.73) [43]. As the behavioral health industry grows, it will be important to consider what entities will hold these organizations accountable for offering a cost-effective service [7, 44]. Multiple available EBIs may help control prices through a competitive market.

#### Success stage

In the success stage of small business growth, companies either create stable practices or focus on creating structures that will allow them to grow [24]. During this stage, marketing is important. Trademark IP (i.e., brand names) can be leveraged to build EBI awareness. Program brand name awareness can help clients, agencies, and providers identify interventions that work. Program trademarks are shorthand for a package of EBIs that can be studied for their efficacy and cost-effectiveness [45]. Ideally, program reputation is based on research evidence in addition to positive consumer experience and marketing. Marketing can include traditional advertising, as well as persuading influencers (e.g., policymakers, professional bodies) and potential referral sources [46]. Brand loyalty can lead to repeat customers and can ease agencies into further EBI adoption [47, 48]. The implementation organizations reported that professional conferences are a strategy to maintain provider brand awareness, visibility, and loyalty. The organizations reviewed believed that name recognition, and related consumer demand and awareness, helped enable them to scale their EBIs nationally and internationally.

Credentialing is one way that implementation organizations can generate value from trademark IP. Accreditation (aka certification) requirements vary for each organization, ranging from attending a training course to multiple tape session reviews by the implementation organization (see Table 2). Some organizations have multiple level of accreditation levels, with higher costs associated with higher levels. Negotiations of standards between the payor and organization are part of contracting, as local standards differ [49]. Regardless of specific requirements, when a funder pays for clinicians to be accredited in an EBI, they assume that client outcomes will improve [22]. Quality assurance mechanisms (e.g., supervision, fidelity monitoring) are important to ensure accreditation translates to improved client outcomes. Accreditation effectively establishes a trademark/service mark (X-certified therapist) licensed to the provider to promote their services as evidence-based. Completing a training and accreditation process has been found to be related to practitioners’ program use [50].

Branding can be misleading. A consumer perception of a brand and the accuracy of the information conveyed by the brand are not necessarily isomorphic. An EBI’s benefit can be decreased when marketing forces are shaped by factors other than program efficacy. Consumers need to differentiate between evidence-based and pseudoscientific brands [51]. EBI registries distill information from hundreds of trials for consumers, but the registries have varied standards for qualifying as an EBI [52]. Some promote empirically supported principles; others promote “branded” therapies [53]. There is a risk that the field and consumers may focus on accentuating brand differentiation when there is little. Even some brands (e.g., MAP) distill common elements of other branded protocols [54]. Given that not all EBIs have overlapping functions or providers (e.g., teachers vs. clinicians), higher level coordination between programs would maximize the benefit of multiple EBIs to the service system (e.g., the International Congress on Evidence-Based Parenting Support hosted by the Parenting and Family Research Alliance).

Advertising and lobbying are not a primary focus of the five organizations reviewed due to their brand recognition. Unlike other industries (e.g., pharmaceuticals) and many behavioral health organizations funded by private capital (e.g., BetterHelp, Talkspace, Cerebral), the five organizations spend few resources on direct-to-consumer marketing. Triple P International developed several social marketing and communication strategies for the Stay Positive public health campaign, which is part of the universal, multitiered Triple P system [55]. However, most contracts are initiated by an interested party (e.g., government agency, schools, hospitals, mental health organizations) who has heard of the program through referrals or word of mouth. Some organizations (e.g., Triple P International and the PAXIS Institute) proactively work with organizations and policymakers to inform them of services; structure systems of adoption, implementation, and maintenance of their programs; and request funding for implementation initiatives. Brand awareness, as well as knowledge of and personal connection to a program developer, may affect policymakers’ funding decisions. If EBI funding decisions and availability are partly based on word of mouth and connections, a challenge for the field is to ensure equitable access to EBIs of people who live in areas without such connections.

It should be noted that the five implementation organizations reviewed rarely work directly with EBI recipients. Triple P International disseminates an online version of Triple P, which can be delivered directly to the consumer. The organizations reported that they improve more equitable access to EBIs by training providers in a variety of settings and levels of care (e.g., schools, primary care, community mental health) that serve underserved communities. Some implementation organizations (e.g., PracticeWise) use evidence-based approaches to address barriers to treatment and to increase their capacity to promote access. However, these efforts do not address structural barriers to mental health care [56, 57], such as cost, language, and distance to treatment facilities. Additional strategies beyond the existence of implementation organizations are needed to improve equitable access to EBIs.

#### Take-off stage

In the take-off stage of small business growth, the business’s reach increases [24]. For implementation organizations, delegation of providing trainings is an important step to increase the number of providers trained in an EBI. Training and ongoing consultation help EBIs be implemented as effectively as in randomized control trials [58]. Although pro bono trainings are admirable, pro bono work is difficult to sustain at scale without government/nonprofit funding (including research grant funding). There may be a trade-off between offering less intensive training to increase program reach, versus offering more intensive training to increase program fidelity. Online trainings can help increase reach, although online trainings are improved with consultation, which may limit reach [58]. Simply granting access to EBIs does not guarantee increased EBI use [58, 59]. Therefore, it is unlikely that free materials with Creative Commons licenses will lead to a meaningful increase in proper EBI use. Thus, less costly implementation support may lead to a loss of fidelity and sustainability [60].

For quality control, IPOs typically require rigorous training. Train-the-trainer models can help increase the supply of trainers [58]. Some implementation organizations limit trainer numbers to maintain quality control and fidelity, and research suggests expert-led trainings are superior to those using train-the-trainer [58]. However, at what point does the focus on quality control lead organizations to become EBI training gatekeepers? Again, the trade-off is between training fidelity and reach. Some organizations set up checks and balances to manage the risk of lower fidelity (e.g., reviewing sessions, limiting train-the-trainer to one generation, requiring recertification), while others require the use of quality assurance/improvement targets.

Implementation consultants help tailor a program to fit local culture, context, and cost constraints while maintaining program fidelity [60]. For example, tailoring the Incredible Years programs to family background, education, and culture as well as child developmental status is an integral part of the Incredible Years accreditation process. *Te Whānau Pou Toru* is a Māori adaptation of Triple P collaboratively created with Māori tribal elders and Triple P program creators [61]. The PAXIS Institute has a process to approve field-initiated modifications to the program consistent with the evidence base. Indeed, a core part of EBI training is to teach providers to implement programs flexibly with fidelity [62].

#### Maturity stage

**Fig. 1:**
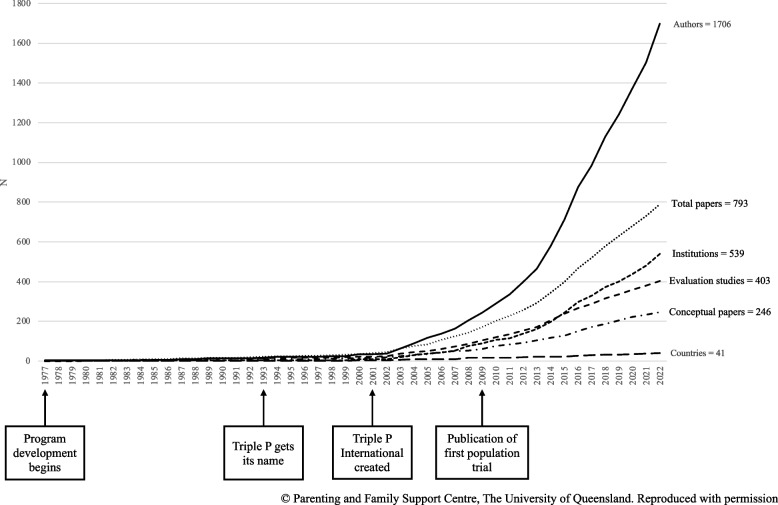
The growth of Triple P research following the creation of Triple P International

In the maturity stage, the business focuses on redesigning structures to account for increased revenue while trying to maintain their entrepreneurial spirit and innovation [24]. Research efforts are especially useful to help implementation organizations stay competitive and avoid becoming obsolete [24]. The five organizations reviewed maintain ongoing research partnerships to foster research-informed developments and independent evaluations. End- users of EBIs and implementation organizations can suggest ideas for research [63, 64]. Researchers are encouraged to be more market facing (i.e., understanding clinicians’ and clients’ needs), rather than being market led (i.e., letting business override research) [65]. The organizations reviewed reported a commitment to evidence and integrity from initial development to evaluation of implementation outcomes. For example, Triple P International experienced consumer demand for a version of Triple P for parents with babies but delayed its release until there was sufficient evidence supporting its efficacy. Researcher partnerships with implementation organizations provide a clear path-to-market for innovations and help EBIs be “designed for dissemination” [5, 64]. Does establishing a purveyor organization decrease EBI research? Using Triple P as an example (Fig. 1), the opposite can occur; increased research activity followed, particularly in growth of independent evaluations.

Collaborations between researchers and implementation organizations can provide students with industry experience. For example, PracticeWise has involved students in community events, secondary data analysis, literature reviews, building and testing assessment and performance projects, building and piloting training materials, attending training and conferences, and strategic planning. These opportunities are valuable given how few training programs provide such experience [66, 67].

Conducting research with commercialized programs is a source of potential conflict of interest (COI). In academia, potential COI may arise from income generated by books, lectures, professional fees, etc. [68]. In the context of implementation organizations, COI potentially occurs because of revenues made from royalties, consultancy fees, and organizations ownership. Potential COI does not inherently corrupt research results, but transparency and disclosure are essential, including on peer-reviewed and invited scientific publications, reports, conferences, websites, grant applications, and trial registries [69, 70]. Researchers who work with our illustrative organizations reported having policies to disclose potential COI [69]. Researchers at universities also complete annual financial disclosure forms. The organizations reviewed reportedly have research integrity policies and procedures to safeguard against potential COI, similar to research universities.

Does involvement with purveyors stifle negative research results? The organizations reviewed reported valuing independent evaluation and implementation efforts to improve practices. For example, in its research agreements, PracticeWise cedes the right of independent authorship, requiring only advanced publication notice to allow time to formulate a response if desired. Many implementation organizations have specific policies to ensure that all results (including null or negative findings) are published wherever possible. Systematic replication and independent evaluation are widely considered the key to becoming recognized as an EBI. However, as implementation organizations are usually involved in training trial clinicians, independent evaluations may still involve some developer or purveyor involvement.

## Conclusions

We identified key themes that affect implementation organization decision-making across the Five Stages of Small Business Growth, including corporate structures, IP licensing agreements, pricing, and balancing fidelity and reach. Franks and Bory [12] found that IPOs are increasingly aware of empirical implementation evidence and understand the concept of implementation science, but many IPO employees are not explicitly trained in D&I. Because implementation organizations are one effective strategy to scale EBIs, evaluations of the facilitators, barriers, and motivators of change within these organizations and the adopting agencies are critical to mitigate a potential gap between implementation research and practice.

To increase EBI reach, research should examine how to make access to programs more affordable, scalable, and effective. There is a need to examine (a) how much and what type of training and ongoing consultation is needed to maximize client outcomes and (b) what type and level of fidelity/program integrity is needed to maximize client outcomes and justify training cost [71, 72]. Is there a differential benefit in training new practitioners relative to *re*training practicing practitioners? It is unclear whether certifications/accreditation is an effective way to increase EBI fidelity in the short and long term. Additional research on mechanisms of change will help organizations refine and improve trainings. For example, online and hybrid trainings, including training using artificial intelligence, could increase EBI reach.

Future research can examine how to coordinate implementation initiatives of IPOs and other implementation organizations within service systems. Which implementation outcomes are the most important? In what ways do IPOs serve as facilitators, and perhaps barriers, to implementation? How do agencies select programs to implement (e.g., personal contact, peer advocacy, social media, or published research)? Knowledge management and decision support may be facilitated through external organizations (e.g., intermediary organizations) or within an organization. Finally, research can examine how to increase consumer’s knowledge of how to seek EBIs.

There are several limitations to this paper that warrant discussion. First, the themes gathered in the manuscript were developed through a co-creation process with the organizations, rather than an independent qualitative analysis (potential bias). Additional themes may have emerged from a different approach. We limited our review to five organizations, which mostly focus on disseminating EBIs for youth and operate primarily from the USA and Australia. Other implementation organizations, including organizations based in other countries, may have different perspectives.

The behavioral health industry is growing, and diverse approaches are used to disseminate EBIs. Future research can examine business models to enhance equitable EBI reach. In the meantime, we expect that in a time of unprecedented need for mental health services, more implementation organizations will be established to help scale existing EBIs.

## Data Availability

Not applicable.

## Appendix

### Survey Questions

1. What does your organization do?
2. How does your organization seek to achieve sustainable dissemination of evidence-based interventions?
3. How you’ve balanced potential conflicts of interest when doing research?
4. Any short comings you see of your model/having purveyor organizations?
5. What would be the one “next needed” study to maximize sustainable implementation of evidence-based interventions in the context of purveyor organizations?
6. How has your organization balanced financial realities of running an organization with making programs affordable to clients?
7. How your organization works with stakeholders, policy makers, community organizations?
8. How your organization provides dissemination and implementation training opportunities that students wouldn’t get from a university setting?
9. Do you see there being a value of the program brand to increasing pull demand and consumer awareness of their program? If so, please describe.
10. Other learnings (successes and failures)?
11. Other key points

## Abbreviations

EBI: Evidence-based intervention
D&I: Dissemination and implementation
IPO: Intermediary and purveyor organization
IP: Intellectual property
B2B: Business-to-business
B2C: Business-to-consumer

## Footnotes

1 We refer to these organizations simply as implementation organization because some authors involved with these organizations either did not think the term “purveyor organization” or “intermediary organization” captured their work (e.g., because they disseminate many–rather than a single–EBIs at an international–not a local–scale); they felt that the term IPO was “too much academic jargon,” or they generally disliked the IPO term.
